# *In-Vivo* Biodistribution and Safety of ^99m^Tc-LLP2A-HYNIC in Canine Non-Hodgkin Lymphoma

**DOI:** 10.1371/journal.pone.0034404

**Published:** 2012-04-24

**Authors:** Allison L. Zwingenberger, Michael S. Kent, Ruiwu Liu, David L. Kukis, Erik R. Wisner, Sally J. DeNardo, Sandra L. Taylor, Xiucui Chen, Kit S. Lam

**Affiliations:** 1 Department of Surgical and Radiological Sciences, School of Veterinary Medicine, University of California Davis, Davis, California, United States of America; 2 Department of Biochemistry and Molecular Medicine, University of California Davis, Sacramento, California, United States of America; 3 Center for Molecular and Genome Imaging, University of California Davis, Davis, California, United States of America; 4 Division of Hematology/Oncology, Department of Internal Medicine, University of California Davis Medical Center, Sacramento, California, United States of America; 5 Clinical and Translational Science Center, School of Medicine, University of California Davis, Sacramento, California, United States of America; Genentech, United States of America

## Abstract

Theranostic agents are critical for improving the diagnosis and treatment of non-Hodgkin Lymphoma (NHL). The peptidomimetic LLP2A is a novel peptide receptor radiotherapy candidate for treating NHL that expresses the activated α4β1 integrin. Tumor-bearing dogs are an excellent model of human NHL with similar clinical characteristics, behavior, and compressed clinical course. Canine *in vivo* imaging studies will provide valuable biodistribution and affinity information that reflects a diverse clinical population of lymphoma. This may also help to determine potential dose-limiting radiotoxicity to organs in human clinical trials. To validate this construct in a naturally occurring model of NHL, we performed *in-vivo* molecular targeted imaging and biodistribution in 3 normal dogs and 5 NHL bearing dogs. ^99m^Tc-LLP2A-HYNIC-PEG and ^99m^Tc-LLP2A-HYNIC were successfully synthesized and had very good labeling efficiency and radiochemical purity. ^99m^Tc-LLP2A-HYNIC and ^99m^Tc-LLP2A-HYNIC-PEG had biodistribution in keeping with their molecular size, with ^99m^Tc-LLP2A-HYNIC-PEG remaining longer in the circulation, having higher tissue uptake, and having more activity in the liver compared to ^99m^Tc-LLP2A-HYNIC. ^99m^Tc-LLP2A-HYNIC was mainly eliminated through the kidneys with some residual activity. Radioactivity was reduced to near-background levels at 6 hours after injection. In NHL dogs, tumor showed moderately increased activity over background, with tumor activity in B-cell lymphoma dogs decreasing after chemotherapy. This compound is promising in the development of targeted drug-delivery radiopharmaceuticals and may contribute to translational work in people affected by non-Hodgkin lymphoma.

## Introduction

The α4β1 integrin is widely expressed on Non-Hodgkin’s Lymphoma (NHL) cells [1], [2], and provides a new target for agents capable of selectively binding to this site. The integrin normally plays a role in lymphocyte homing, and passage of the lymphocytes through the endothelial wall, to sites of inflammation, and may contribute to dissemination of NHL [3], [4]. It has recently been identified as a target for both imaging and treatment of relapsed and refractory NHL using imaging dosimetry of the therapeutic target, CD20 [5]. However, new targeted agents are needed for first line therapy of NHL by a high affinity peptidomimetic ligand. The peptidomimetic LLP2A(*N*-[[4-[[[(2-ethylphenyl)amino]carbonyl]amino]phenyl]acetyl]-*N* ^ε^-6-[(*2E*)-1-oxo-3-(3-pyridinyl-2-propenyl)]-L-lysyl-L-2-aminohexanedioyl-(1-amino-1-cyclohexane)carboxamide), is comprised of D- and unnatural amino acids that are resistant to proteases in human plasma. Theranostic agents are critical for improving the diagnosis and treatment of non-Hodgkin Lymphoma (NHL). NHL is a very radiosensitive tumor making it ideal for development of targeted radiotherapeutic drugs. Radioimmunotherapy (RIT) has revolutionized the imaging and treatment of relapsed and refractory NHL using imaging dosimetry of the therapeutic target, CD20 [5]. However, new targeted agents are needed for first line therapy, patients who do not respond to currently available therapies, and for those who relapse after RIT. Reversible bone marrow suppression is the dose limiting toxicity for Y-90 Ibrutumomab tiuxetan and I-131 tositumomab [6]. RIT with MAb to other target provide therapeutic alternatives as well but marrow suppression continues to be the limiting toxicity [7]. Peptide receptor radiation therapy (PRRT) is an alternative strategy for delivering diagnostic and therapeutic agents to tumors [8]. The peptidomimetic LLP2A (*N*-[[4-[[[(2-ethylphenyl)amino]carbonyl]amino]phenyl]acetyl]-*N* ^ε^-6-[(*2E*)-1-oxo-3-(3-pyridinyl-2-propenyl)]-L-lysyl-L-2-aminohexanedioyl-(1-amino-1-cyclohexane)carboxamide), is comprised of D- and unnatural amino acids that are resistant to proteases in human plasma [9]. LLP2A has a high affinity to activated α4β1 on lymphoma cells, and the integrin has been found to be important in tumor growth and metastasis [4]. LLP2A is a novel PRRT candidate for treating NHL that expresses the activated α4β1 integrin [9]. LLP2A is a novel PRRT candidate for treating NHL that expresses the activated α4β1 integrin [9].

Theranostic agents are critical for improving the diagnosis and treatment of Non-Hodgkin’s Lymphoma (NHL) [3]. NHL is a very radiosensitive tumor making it ideal for development of targeted radiotherapeutic drugs. Radioimmunotherapy (RIT) has revolutionized the imaging and treatment of relapsed and refractory NHL using imaging dosimetry of the therapeutic target, CD20 [5]. However, new targeted agents are needed for first line therapy, patients who do not respond to currently available therapies, and for those who relapse after RIT. Reversible bone marrow suppression is the dose limiting toxicity for Y-90 Ibrutumomab tiuxetan and I-131 tositumomab [6]. RIT with MAb to other target provide therapeutic alternatives as well but marrow suppression continues to be the limiting toxicity [7]. Peptide receptor radiation therapy (PRRT) is an alternative strategy for delivering diagnostic and therapeutic agents to tumors [8].

Nanotechnology theranostics have been developed as a multi-faceted approach to countering the intratumor and biological intertumor variability found in cancer patients. Ligands are often used to provide targeting of the carrier to the designated site, such as small molecules, peptides, peptidomimetics, carbohydrates, monoclonal antibodies, and DNA/RNA aptamers [10]. The use of smaller particles less than 100 nm will reduce clearance by the reticuloendothelial system [11]. We aimed to develop an agent with theranostic potential that is small enough in size to penetrate into cells, and has biodistribution properties that allow a good therapeutic index. ^99m^Tc-LLP2A-HYNIC, comprised of peptidomimetic and radiopharmaceutical, is very small (<1 nM), which allows it to overcome many of these obstacles to tumor targeting.

Targeted therapies are hindered in translation from the bench to the clinic by pre-clinical studies that do not accurately represent the range of conditions found in naturally occurring tumors in immune competent hosts. Tumor-bearing dogs are an excellent model of human NHL with similar clinical characteristics, behavior, and compressed clinical course [12], [13]. The natural inhomogeneity of the canine model and rapid progression of disease allow realistic testing of the behavior of a new drug *in vivo*. The biological variations in expression of integrins are also more likely to reflect the clinical situation than those of cell lines. LLP2A has been tested *in vitro* using canine cell lines and tissue samples from affected animals, and has been found to have good affinity to canine lymphoma, although less than that reported in cell lines and xenografts of human cell lines in mice [9], [14], [15]. Constructs including ^111^In-LLP2A-DOTA, and optical imaging probes (LLP2A-Cy5.5) have been used to confirm affinity of LLP2A to xenografts in mice [14], [16]. Canine *in vivo* imaging studies will provide valuable biodistribution and affinity information that reflects a diverse clinical population of lymphoma. This may also help to determine potential dose-limiting radiotoxicity to organs in human clinical trials.

To validate this construct in a naturally occurring model of NHL, we performed *in-vivo* molecular targeted imaging and biodistribution in 3 normal dogs and 5 NHL bearing dogs. From discovery of the optimum high-affinity peptide for naturally occurring tumors, to data closely paralleling a human phase 0/1 clinical trial, this study provides baseline information for further development of LLP2A as a new theranostic agent. The safety and toxicity information in tumor-bearing dogs, and dosimetry predictions for trials of the radiotherapeutic agent, will be extremely valuable in planning human clinical trials. A novel PRRT therapy could improve the treatment response of NHL patients with high expression of the α4β1 integrin.

## Materials and Methods

### Preparation of LLP2A-HYNIC

#### Synthesis of LLP2A-HYNIC

**Figure 1:**
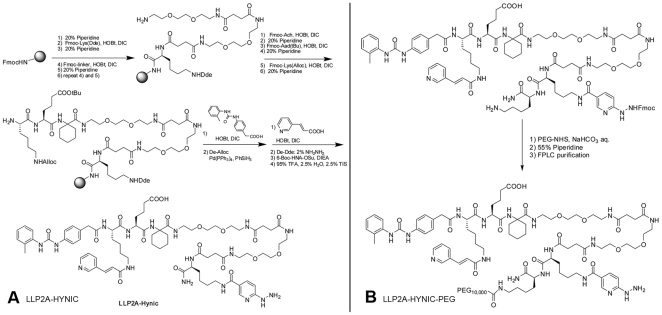
Synthesis of LLP2A-HYNIC and LLP2A-HYNIC-PEG. A. Synthetic approach for LLP2A-HYNIC. B. Synthetic approach of LLP2A-HYNIC-PEG.

LLP2A-HYNIC was designed to have HYNIC attached to the side chain of Lys, and two hydrophilic linkers betweenLLP2A and Lys(HYNIC). The synthetic scheme is shown in Figure 1A. The synthesis was performed on rink amide MBHA resin using a similar approach as we previously reported [14]. 6-Boc-HNA-OSu (3 eq. to resin, from Solulink, Inc, San Diego, CA) was coupled to the ε-amino group of lysine in presence of diisopropylethylamine (6 eq.). The cleaved crude products were purified by semi-preparative reversed-phase high performance liquid chromatography (RP-HPLC) and lyophilized to give a white powder. The purity was determined to be >95% on analytical HPLC. The identity of the compounds was confirmed by Matrix-assisted laser desorption/ionization time of flight mass spectrometry (MALDI-TOF MS): [M+Na^+^]: 1556.78 (calculated: 1556.79).

#### Preparation of LLP2A-HYNIC-PEG conjugate

A D-Lys was added to the *C*-terminal of LLP2A-HYNIC, therefore its ε-amino group could be used as the site for PEGylation (Figure 1B). The synthesis of LLP2A-HYNIC(Fmoc)-D-Lys is similar to LLP2A-HYNIC except an additional Fmoc-D-Lys(Boc) as the first amino acid was attached to the rink amide resin, and 6-Fmoc-HNA-OSu (Solulink, Inc, San Diego, CA) was used to replace 6-Boc-HNA-OSu. 10,000-Da PEG was selected according to a previous study [14]. PEGylation was achieved with 10,000-Da mPEG-succinimidylα-methylbutanoate (PEG-NHS ester, Nektar Therapeutics AL Corporation, Huntsville, AL) using the same approach as we previously reported [14].

#### Radiopharmaceutical preparation–^99m^Tc-LLP2A-HYNIC

In a typical reaction, [^99m^Tc]technetium pertechnetate (^99m^TcO_4_; GE Healthcare, Sacramento, CA; 29 mCi) in saline (150 µL) was combined with 1 M NH_4_OAc, (pH 5.2, 50 µL), LLP2A-HYNIC (MW 1534; 0.45 mg, or 290 nmol) in 0.25 M NH_4_OAc, pH 5.2 (45 µL), tricine (4.5 mg) in H_2_O (45 µL), and SnCl_2_•2H_2_O (25 µg) in 10 mM HCl (5 µL). The solution was incubated on a heating block at 60–70°C for 1 hour, and purified by C18 solid phase extraction (SPE; ORTG, Oakdale, TN). Purified ^99m^Tc-LLP2A-HYNIC (20 mCi) in 1∶1 (v/v) ethanol:saline eluent (0.25 mL) was formulated with unlabeled LLP2A-HYNIC and PBS to a final concentration of 2 mCi/0.2 mg/mL, sterilized by 0.22 µm filtration, and dosed at 0.05 mg (33 nmol) per kg body weight. Thin layer chromatography (TLC) was performed to evaluate radiochemical purity of the product.

#### Radiopharmaceutical preparation–^99m^Tc-LLP2A-HYNIC-PEG

LLP2A-HYNIC-PEG (MW 12,095) was radiolabeled similarly, and dosed at 0.4 mg (33 nmol) per kg body weight.

### Animal Care and Use

This study was carried out in strict accordance with the recommendations in the Guide for the Care and Use of Laboratory Animals by the National Research Council. This study was conducted with the approval of the University of California, Davis Animal Care and Use Committee, protocol 10806 and 12349. All efforts were made to minimize stress during imaging.

### Safety and Toxicity Assays in Mice

Before initiating *in vivo* tests in dogs using LLP2A-HYNIC, an *in vivo* study using mice was conducted to assess safety. LLP2A-HYNIC was injected intraperitoneally into mice at 2 doses (10 µg/mouse and 100 µg/mouse). There were 5 and 6 mice in each treatment, respectively. Mice were weighed daily for 1 week. Pooled blood samples were collected at 5 day intervals until 20 days post injection. For the mouse safety study, weight was modeled as a function of time with an autoregression correlation structure to account for correlation of measurements across time. To account for varying starting weights among mice, each mouse’s starting weight was subtracted from subsequent weights and the model was fit without an intercept. These analyses were conducted using Proc Mixed in SAS Version 9.2. The pooled platelet count and white blood cell count were graphically examined.

### Biodistribution

#### Imaging equipment and software

All dogs were imaged with a Gamma camera (Technicare Omega 500, Solon, Ohio, or IS2, Ottawa, Ontario). Images were obtained of the whole body in overlapping segments, in right lateral, left lateral, and sternal recumbency. Images were obtained to 200,000 counts, with a 256×256 bit matrix and 16-bit depth. A camera standard of 200 uCi in 10 mL of water was included in each image to ensure stable measurements over the scan period. Camera sensitivity was determined by placing the camera standard 30 cm from the detector and scanning for 1 minute. This calibration factor was used to convert individual organ emission counts to mCi.

#### Normal dog biodistribution

All 3 dogs were mixed breed dogs and after physical examination were clinically normal. The 3 normal dogs were imaged using both ^99m^Tc-LLP2A-HYNIC and ^99m^Tc-LLP2A-HYNIC-PEG, given 24 hours apart, to determine normal biodistribution and imaging characteristics of the agents. The labeled peptide (10 mCi on 0.05 mg/kg ^99m^Tc-LLP2A-HYNIC or 0.4 mg/kg ^99m^Tc-LLP2A-HYNIC-PEG) was injected by slow bolus into a catheterized cephalic vein, and the dogs were monitored for adverse reactions in heart rate, respiratory rate, temperature, and blood pressure at 0, 15, 30, 60, 120, and 240 minutes. Images of the whole body in orthogonal planes were acquired at 2 hours, 4 hours, and 6 hours after injection. Complete blood counts and serum biochemistry were performed before and after imaging.

#### Imaging of dogs affected with lymphoma

5 dogs with naturally occurring lymphoma were imaged using the above protocol. Three dogs had B-cell lymphoma and 2 dogs had T-cell lymphoma. Dogs were staged by cytologic evaluation of fine needle aspirates of the single affected lymph node, or a representative lymph node in diffuse disease. Abdominal ultrasound, bone marrow aspirates, and thoracic radiographs were also performed. ^99m^Tc-LLP2A-HYNIC was used in 2 dogs with B-cell lymphoma, 1 of which was imaged before and after the initial course of chemotherapy (9 weeks). ^99m^Tc-LLP2A-HYNIC-PEG was used in 3 dogs, 1 B- and 2 T-cell lymphoma, two of which were imaged both before and after chemotherapy (6 weeks and 8 weeks). Chemotherapy consisted of prednisone, vincristine, cyclophosphamide, and L-asparaginase. Two dogs had whole body computed tomography (CT) performed. One dog had pre- and postcontrast (Isovue 370, Bracco Diagnostics Inc., Princeton, NJ) CT imaging pretreatment and post-treatment, and one dog had non-contrast enhanced CT imaging performed pretreatment. CT scans were performed at 120 kVp and 150 mA with 7 mm collimation using a helical scanner (GE Prospeed, General Electric Co., Milwaukee, WI).

#### Whole body biodistribution

Blood samples were obtained from all dogs at time 0, 15, 30, 60, 120, 240, and 360 minutes to document clearance of the radiopharmaceutical. 2 mL of blood were drawn into a heparinized syringe. Aliquots of 0.5 mL samples were processed with a well counter (PerkinElmer Wizard 1470, Covina, California) calibrated to ^99m^Tc. The radioactivity in blood was calculated by using total blood volume based on body weight, and was decay-corrected [17]. In addition, radioactivity readings of the whole body were taken using a handheld monitor at 1 m distance from the dog at time 0, 15, 30, 60, 120, 240, 360 minutes, and 24 hours.

All dogs were held overnight in a protected area with institutionally approved radiation safety protocols. Dogs were released from the facility after whole body radiation levels were less than 0.5 mR/hr at the skin surface and less than 0.05 mR/hr at 1 m.

#### Organ uptake of LLP2A

For all scintigraphic studies, regions of interest (ROI) were drawn around each organ of interest (liver, lung, bone marrow, lymph node, gastrointestinal tract, kidney, salivary gland) on images acquired at 2 hours, 4 hours, and 6 hours after injection. ROIs were drawn on the 2 hour images and duplicated for the opposite lateral projection and subsequent time points, and adjusted for altered patient position. ROIs were also drawn in the temporal muscle of the head (background correction for lymph nodes and salivary gland), longissimus thoracis muscles (background correction for bone marrow activity in the thoracic spine), the iliocostalis muscles (background correction for liver and kidney), and combined abdominal musculature (background correction for gastrointestinal tract). The counts and pixels of each ROI were obtained using imaging software (Nuclear Mac, Scientific Imaging Inc., Crested Butte, CO; or Osirix Imaging Software, Version 2.7.5; http://www.osirix-viewer.com/).

Organs that were clearly delineated on conjugate projections (liver, GI, bone marrow, lung) were measured using the geometric mean method, and smaller or peripherally located organs (kidney, mandibular lymph nodes, salivary gland) were measured using the effective point source method [18]. Depth for depth correction was measured on orthogonal images. Lung activity was measured using an ROI in a posterior region and normalized to the total pixels of the lung [19]. Attenuation correction was achieved using the equation for ^99m^Tc in soft tissue [20]. Background correction was used in all calculations [18]. Lung activity was measured using an ROI in a posterior region and normalized to the total pixels of the lung [19]. Attenuation correction was achieved using the equation for ^99m^Tc in soft tissue [20]. Background correction was used in all calculations. Camera sensitivity was measured using the camera standard. Percent injected dose and organ:muscle ratio were calculated for each imaging study at each time point. Imaging results are presented in graphical format as percent injected dose for each organ evaluated, as well as organ:muscle ratios.

Blood levels (percent injected dose) over time are provided in Table S1. Whole body activity (mR/hr) data is in Table S2 and the biodistribution data are provided in Table S3. The specific activity of each dose is provided in Table S4.

## Results

### Safety and Toxicity Assay in Mice

**Figure 2:**
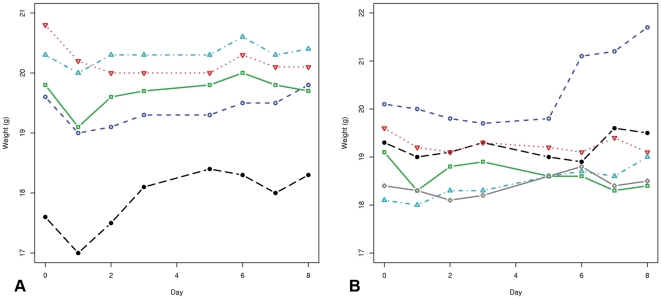
Safety assay of LLP2A-HYNIC in mice. A. Weight (g) of five mice treated with 10 µg/100µL LLP2A-HYNIC over 8 days. B. Weight (g) of six mice treated with 100 µg/100µL LLP2A-HYNIC over 8 days.

At the 10 µl/mouse dosage, weight significantly differed across days (F_7,13_ = 3.86, p = 0.017). Weight significantly decreased the first day after injection (*t* = −3.09, p = 0.016). However, after the first day, weight increased and did not differ significantly from the starting weight on any subsequent day (Figure 2A). At the 100 µl/mouse dosage, weight did not differ from the starting weight at any time (F_7,14_ = 0.78, p = 0.616; Figure 2B). White blood cell counts and platelet counts also remained within normal ranges until day 20 (data not shown).

#### Radiopharmaceutical preparation

^99m^Tc-LLP2A-HYNIC and ^99m^Tc-LLP2A-HYNIC-PEG were produced with mean yields of 67 (±10)% and 30 (±8)%, respectively, and the radiochemical purity of every dose was >95%. The mean injected dose per dog was 7.7 mCi (range 2.8–10.7 mCi), or 0.41 mCi/kg (range 0.15–0.84). The mean specific activity of ^99m^Tc-LLP2A-HYNIC injected was 8.95 mCi/mg (SD 2.38), and of ^99m^Tc-LLP2A-HYNIC-PEG was 1.29 mCi/mg (SD 0.59).

### Biodistribution

#### Blood and Whole body activity

Both ^99m^Tc-LLP2A-HYNIC and ^99m^Tc-LLP2A-HYNIC-PEG had high activity, with the pegylated form having a greater whole body activity initially. The mean whole body activity of ^99m^Tc-LLP2A-HYNIC at 5 minutes post-injection was 0.5 mR/h (range 0.2–0.8), and the mean whole body activity of ^99m^Tc-LLP2A-HYNIC-PEG was 1.1 mR/h (range 1–1.5). At 360 min the mean whole body activity of ^99m^Tc-LLP2A-HYNIC had decreased to 0.13 mR/h (range 0.03–0.05) and that of ^99m^Tc-LLP2A-HYNIC-PEG was 0.19 mR/h (range 0.02–0.4). By 24 hours the activities were similar for the nonpegylated and pegylated forms (0.04 and 0.05 mR/h, respectively).

**Figure 3:**
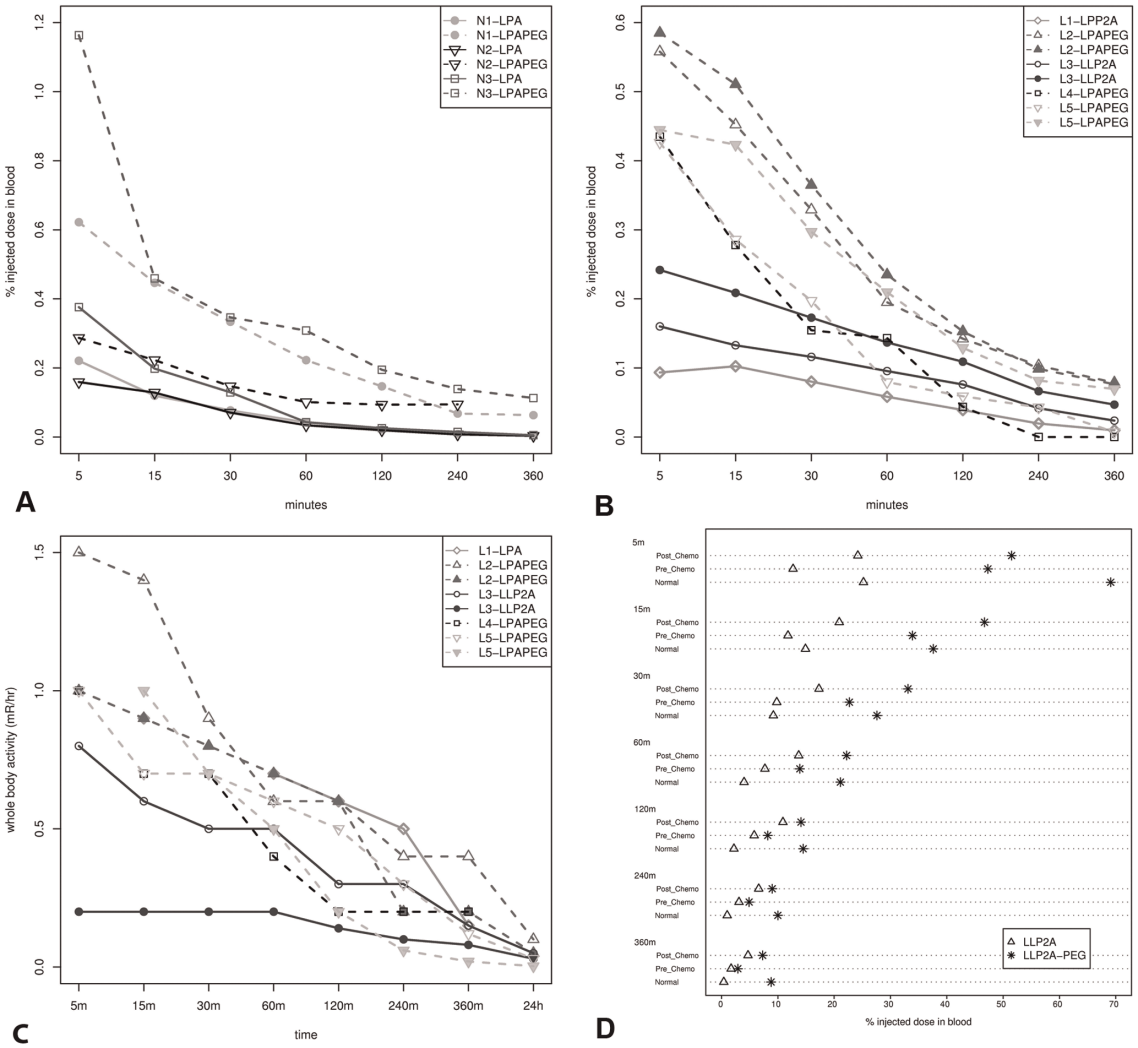
Blood clearance of ^99m^Tc-LLP2A-HYNIC and ^99m^Tc-LLP2A-HYNIC-PEG in dogs. Normal dogs (N1–N3) had higher % injected dose (PID) (A) than those with lymphoma (L1–L5) (B). In B and C, open symbols represent pre-chemotherapy and closed symbols represent post-chemotherapy status. In general ^99m^Tc-LLP2A-HYNIC-PEG had higher blood radioactivity than ^99m^Tc-LLP2A-HYNIC. (C). Whole body dose of dogs with lymphoma at 1 m declined to background levels by 24 hours after administration. (D). Mean Percent injected dose (PID) of ^99m^Tc-LLP2A-HYNIC (triangles) and ^99m^Tc-LLP2A-HYNIC-PEG (stars) in blood over time for pre- and post-chemotherapy lymphoma dogs and in normal dogs.

The Percent Injected Dose (PID) in the blood declined over time in both normal (Figure 3A) and NHL (Figure 3B) dogs. The trajectory of the decline was similar in both groups. Normal dogs sequentially received each LLP2A form. For these dogs, ^99m^Tc-LLP2A-HYNIC-PEG consistently remained in the blood at higher levels than the form without PEG for a given dog (Figure 3A). NHL dogs received only one of the two LLP2A forms thus precluding a paired comparison of the PID trajectories of each form. Nevertheless, NHL dogs receiving the ^99m^Tc-LLP2A-HYNIC-PEG generally had higher PID levels than those receiving the ^99m^Tc-LLP2A-HYNIC form (Figure 2). This difference was most apparent in the first hour after injection.

The activity of one form of LLP2A was evaluated in three dogs (L2, L3, L5) before and after chemotherapy treatment. Blood PID was higher in these dogs after chemotherapy (Figure 3B). The one exception was with L2 at 240 and 360 minutes after injection; PID was nearly equivalent at these times before and after chemotherapy. Whole body activity also declined over time but the patterns observed for blood PID were less evident (Figure 3C). Whole body activity was less than 0.4 mR/hr at 6 hours post injection.

Finally, we compared mean blood PID for ^99m^Tc-LLP2A-HYNIC and ^99m^Tc-LLP2A-HYNIC-PEG over time in normal, pre-chemotherapy and post-chemotherapy dogs (Figure 3D). Mean PID was consistently higher with ^99m^Tc-LLP2A-HYNIC-PEG than ^99m^Tc-LLP2A-HYNIC for all dogs. For ^99m^Tc-LLP2A-HYNIC, post-chemotherapy dogs generally had higher PID than normal or pre-chemotherapy dogs, but this generalization did not hold for ^99m^Tc-LLP2A-HYNIC-PEG.

#### Imaging biodistribution

**Figure 4:**
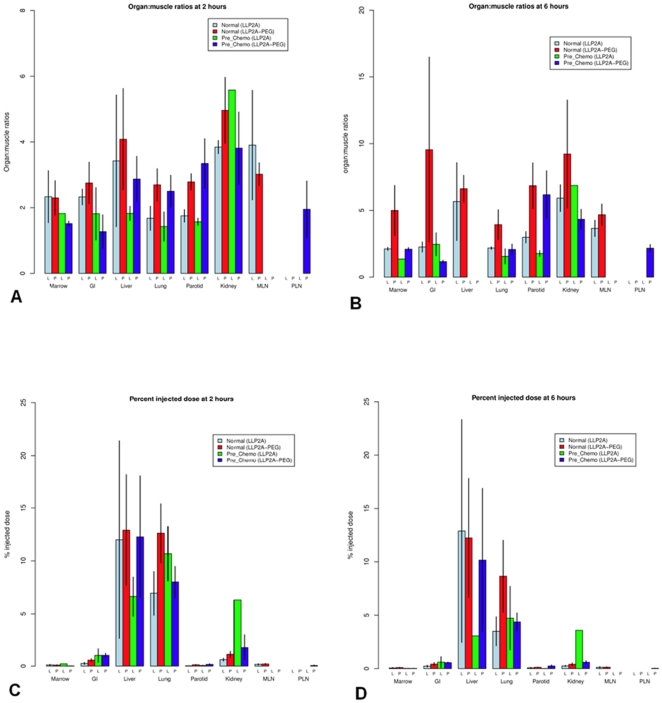
Biodistribution patterns of ^99m^Tc-LLP2A-HYNIC and ^99m^Tc-LLP2A-HYNIC-PEG in normal dogs and dogs with lymphoma (unaffected tissues) prior to chemotherapy treatment. Mean(±SE) organ:muscle ratios of ^99m^Tc-LLP2A-HYNIC and ^99m^Tc-LLP2A-HYNIC-PEG at 2 hours post injection (A) and 6 hours post injection (B).Percent injected dose (PID) of ^99m^Tc-LLP2A-HYNIC and ^99m^Tc-LLP2A-HYNIC-PEG at 2 hours post injection (C) and 6 hours post injection (D). Gastrointestinal tract (GI), mandibular lymph node (MLN), popliteal lymph node (PLN).

Organ:muscle ratios of organs in normal dogs at 2 hours were slightly higher than those in NHL dogs (Figure 4A). Mean(±SE) organ-muscle ratios and percent injected dose in organs at 2 and 6 hours after injection had slightly higher uptake in tissues, particularly the lung, parotid salivary gland, and kidney. At 6 hours, more ^99m^Tc-LLP2A-HYNIC-PEG remained in the tissues compared to ^99m^Tc-LLP2A-HYNIC including the bone marrow, GI tract, parotid salivary gland, and kidney (Figure 4B). The highest PID for both forms of the peptide at 2 and 6 hours were the liver and lung, which represented the largest organs (Figure 4C, 4D). ^99m^Tc-LLP2A-HYNIC-PEG had higher activity in the lung at both time points, which in part likely reflected the higher residual activity in the blood.

NHL dogs had slightly lower organ:muscle ratios at 2 hours, and more significantly lower ratios at 6 hours. This trend was less evident in PID where the normal and NHL dogs were more similar across organs. NHL dogs had a similar increased organ:muscle ratio with ^99m^Tc-LLP2A-HYNIC-PEG than ^99m^Tc-LLP2A-HYNIC except for in the kidneys.

**Figure 5:**
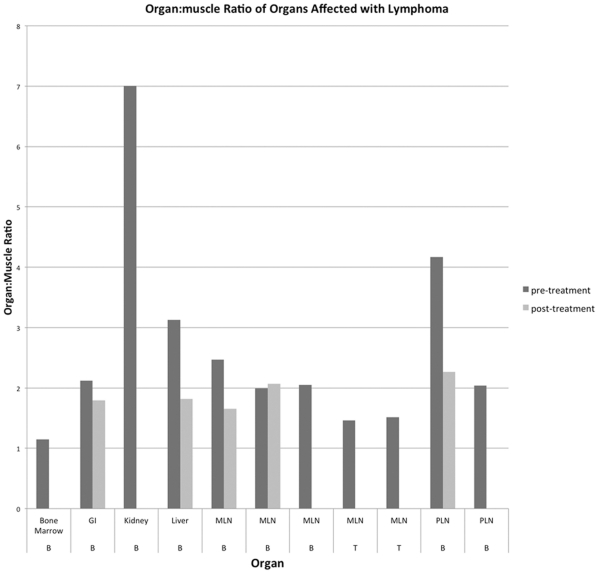
Biopsy proven tissues affected with lymphoma imaged with ^99m^Tc-LLP2A pre- and post-chemotherapy. Tissues have increased activity over background, primarily in the large cell B-cell lymphoma dogs. Activity in these tissues decreased post-chemotherapy. Tissues without a post-treatment measurement were not available for sampling. Gastrointestinal tract (GI), mandibular lymph node (MLN), popliteal lymph node (PLN), B-cell lymphoma (B), T-cell lymphoma (T).

**Figure 6:**
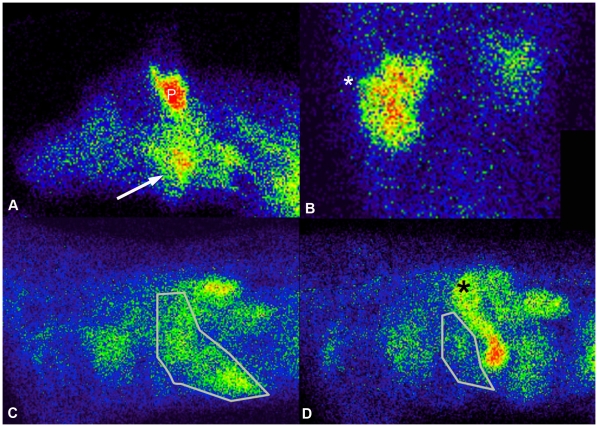
Imaging of dogs with lymphoma using ^99m^Tc-LLP2A-HYNIC-PEG and ^99m^Tc-LLP2A-HYNIC. A. Lateral image of the head at 4 h. Mandibular lymph node activity (arrow) in a dog with lymphoma imaged with ^99m^Tc-LLP2A-HYNIC-PEG. The parotid salivary gland (P) has normal uptake. B. Ventrodorsal projection of the mid-abdomen with head at top of image at 4 h. Renal uptake (white *) in a kidney affected with lymphoma using ^99m^Tc-LLP2A-HYNIC. C. Lateral pre-chemotherapy of the liver (outlined) of the same dog as in A, with head to the left. D. Post chemotherapy, the liver is smaller and has less activity. There is activity visible in the stomach (black *).

Each dog had tissue sampling performed by fine needle aspirate, biopsy, or excision and samples were reviewed by a veterinary pathologist. Tissues in each dog that were suspected to be affected by NHL were sampled, in addition to a representative lymph node (mandibular lymph node) in diffuse disease. The tissues proven to contain lymphoma included bone marrow (L1), gastrointestinal lymph nodes (L2), kidney (L1), liver (L2), mandibular lymph node (L1–5), and retropharyngeal lymph node (L1, L2) (Figure 5). In dogs L4 and L5, the mandibular lymph node was the only site of T-cell lymphoma. Images were compared with affected tissues and the percent injected dose was calculated. Alternate imaging confirmation of affected tissues was performed for affected gastrointestinal lymph nodes, kidney, liver, and retropharyngeal lymph nodes (ultrasonography), and two dogs with enlarged mandibular lymph nodes (CT). B-cell lymphoma affected tissues had higher activity compared to background compared with T-cell lymphoma (Figure 6A). Activity decreased in most tissues post-chemotherapy compared to pre-chemotherapy. The highest organ:muscle ratio (7.00) occurred in a kidney affected with lymphoma (Figure 6B). This activity was higher than in the unaffected kidney in the same dog (5.27). The affected liver also decreased in activity post-chemotherapy (Figure 6C, 6D).

Incidental uptake was seen in the parotid salivary gland in all dogs. Two affected dogs injected with ^99m^Tc-LLP2A-HYNIC-PEG had uptake in the stomach. Serum samples taken from these dogs during the study were tested using HPLC and verified that the radiopharmaceutical had >95% purity.

Heart rate, temperature, respiratory rate, and mucus membrane color of dogs did not change significantly during the period after radiopharmaceutical injection. Two dogs receiving the pegylated form of LLP2A had mild, transient increases in mean blood pressure at 5 and 15 minutes post-injection. One dog with T-cell lymphoma had leukopenia and anemia after the second imaging time point. One dog with B-cell lymphoma had slight progression of increased BUN and creatinine, and one had a mild increase in liver enzymes.

## Discussion

^99m^Tc-LLP2A-HYNIC-PEG and ^99m^Tc-LLP2A-HYNIC were successfully synthesized and had acceptable labeling efficiency and excellent radiochemical purity. ^99m^Tc-LLP2A-HYNIC and ^99m^Tc-LLP2A-HYNIC-PEG had biodistribution in keeping with their molecular size, with ^99m^Tc-LLP2A-HYNIC-PEG remaining longer in the circulation, having higher tissue uptake, and having more activity in the liver compared to ^99m^Tc-LLP2A-HYNIC. ^99m^Tc-LLP2A-HYNIC was mainly eliminated through the kidneys with some residual activity. Radioactivity was reduced to near-background levels at 6 hours after injection. In NHL dogs, tumor showed moderately increased activity over background, with tumor activity in B-cell lymphoma dogs decreasing after chemotherapy.

The slight variability in labeling efficiency resulted in slightly varying total doses of activity to each dog, however the activity was more than sufficient for imaging. The total body dose decayed and cleared in 24 hours providing a practical time frame for imaging as well as the release of the animal from radiation protected facilities. There was uptake of the compounds into the parotid salivary gland in all dogs, and in the stomach of two dogs. When imaging with ^99m^TcO4-, these tissues uptake the radionuclide because of its negative charge. The increased signal in these organs may have been due to small amounts of ^99m^TcO4-, although 95% was bound according to HPLC testing of the pre-injection dose, and in the serum of the two dogs with gastric uptake, indicating that there was little dissociation of ^99m^Tc from the LLP2A *in vivo*. No abnormalities of the stomach were detected on abdominal ultrasound. Free ^99m^TcO4- expression of the target in stomach and salivary glands would be less likely, however has not been studied. A target mediated drug disposition may have been further studied with a dose-escalation study, however additional dogs with NHL were not available.

Both forms of LLP2A had good bioavailability with adequate blood activity and minor tissue activity in normal organs. As expected, the addition of 10,000 MW PEG to the compound increased bioavailability by increasing the size of the molecule and preventing rapid clearance through the renal system [21], [22]. This was evidenced by the higher circulating blood levels compared to the non-pegylated form. There was also some increased activity in the tissues of dogs injected with ^99m^Tc-LLP2A-HYNIC-PEG, which is likely due to increase in charge of the molecule [14], as well as the possibility of increased non-specific binding. In NHL dogs, there was less activity of each form in tissues compared to normal dogs, and a trend toward more activity in the blood. Alterations of lymphocytes in NHL dogs could include increased activation due to inflammation, and leukopenia secondary to chemotherapy. LLP2A has mild affinity to normal lymphocytes which increases with activation [9], [15]. Increased activity in the blood of NHL dogs could be caused by non-specific binding of activated leukocytes, since no dog had documented leukemia at the time of imaging. This in turn could reduce the bioavailability of the compound to tissues.

There was moderate normal tissue uptake of ^99m^Tc-LLP2A-HYNIC-PEG in the bone marrow, GI tract, liver, lung, salivary gland, kidney, and mandibular lymph node. This was less apparent with ^99m^Tc-LLP2A-HYNIC where the liver, kidney, and mandibular lymph node had a lesser degree of uptake. Uptake in normal tissues was less pronounced in the affected dogs in all organs except the kidney, which is the major route of excretion of the radiopharmaceutical. The increased activity in the kidney at 6 hours compared to other organs likely indicates a degree of binding to the renal tubules. This may be caused by the charge of the radiopharmaceutical, which in PEGylated forms has been shown to increase renal and hepatic retention [23]. The activity in the GI tract also indicates that biliary excretion may occur, however this was less apparent in affected dogs.

The spleen is an organ commonly affected by lymphoma in dogs, as was found in all B-cell lymphomas included in this study. However, the spleen was not visible on any image and precluded its inclusion in the measurements. The canine spleen is a long, flat organ, that when imaged in lateral recumbency is not in a position that can overcome the background of liver and GI tract. Radiographs of the abdomen were used to correlate the positions of organs on the scintigraphic images, and a discrete signal was not appreciated.

Tumor-affected tissues had increased activity compared to most normal organs and compared to background signal. Three dogs with high grade B-cell lymphoma showed higher activity compared with the two dogs with intermediate and indolent T-cell lymphoma. LLP2A has been shown to target T-cell lymphoma in human cell lines and xenografts. There may be a species difference causing reduced affinity of LLP2A to canine T-cell lymphoma. There may also be an effect of lower grade tumors expressing less activated alpha4-beta1 integrin on the cell surface.

The kidney affected with lymphoma had the highest activity of the tumor tissues, and was increased compared to the contralateral kidney in the same dog (L1). The radiopharmaceutical used was ^99m^Tc-LLP2A-HYNIC, which tends to have higher renal activity than ^99m^Tc-LLP2A-HYNIC-PEG. The second dog receiving ^99m^Tc-LLP2A-HYNIC with unaffected kidneys (L3) had similar renal activity to the normal kidney in L1. Although the renal PID for this form was higher than ^99m^Tc-LLP2A-HYNIC-PEG, the tumor affinity was higher still.

The mild changes in blood pressure and lack of other change in vital signs indicate safety in the peri-injection period. The dogs with mild changes on CBC and biochemistry were receiving chemotherapy concurrently, making it difficult to attribute changes to the LLP2A constructs. The normal dogs had no changes in blood parameters. More data may be needed to detect any adverse effects, however there were no significant acute or chronic changes.

Affinity to tissues previously affected by NHL to ^99m^Tc-LLP2A-HYNIC and ^99m^Tc-LLP2A-HYNIC-PEG decreased post-chemotherapy. This supports the specificity of the radioligand to canine lymphoma and the alpha4-beta1 integrin. The peptidomimetic has the potential to provide targeted drug delivery to tumor, as well as serving as an imaging marker of response to therapy. As has been done with other radiopharmaceuticals, a test dose may be administered to verify tumor affinity to the ligand and to estimate tumor and whole body dose [24]. To date, this has not been performed in veterinary medicine, in part because of a lack of MIRD phantom for canine organs, which may become possible in the future [25].

In summary, ^99m^Tc-LLP2A-HYNIC-PEG and ^99m^Tc-LLP2A-HYNIC are safe in normal dogs and those with NHL. The radiopharmaceutical is capable of targeting high grade B-cell canine lymphoma-affected tissue, and affinity decreases post-chemotherapy. This compound is promising in the development of targeted drug-delivery radiopharmaceuticals and may contribute to translational work in people affected by Non-Hodgkin lymphoma.

## Supporting Information

Table S1 Percent injected dose of LLP2A or LLP2A-PEG in blood of normal and HLM dogs at 5, 15, 30, 60, 120, 240, and 360 minutes post injections.
(XLSX)

Table S2 Whole body activity (mR/hr) of LLP2A and LLP2A-PEG at 5, 15, 30, 60, 120, 240, and 360 minutes and 24 hours post injection in HLM dogs.
(XLSX)

Table S3 Organ ratios and percent injected dose in study animals at 2, 4 and 6 hours.
(XLSX)

Table S4 Specific activity, injected activity, and injected mass of injected compound of normal dogs N1-N3, and NHL dogs L1-L5.
(DOCX)
