# Supplementary material for: In-Vivo Biodistribution and Safety of 99mTc-LLP2A-HYNIC in Canine Non-Hodgkin Lymphoma
Source: PLoS One. 2012 Apr 24;7(4):e34404. doi: 10.1371/journal.pone.0034404 (PMC3335845; doi:10.1371/journal.pone.0034404)
Supplement: Table S4 — Specific activity, injected activity, and injected mass of injected compound of normal dogs N1-N3, and NHL dogs L1-L5. (DOCX) [file pone.0034404.s004.docx]

|  | **Specific Activity (mCi/mg)** | **Injected activity (mCi)** | **Injected mass (mg)** | **Injected mass (µmol)** |
| --- | --- | --- | --- | --- |
| **^99m^Tc-LLP2A-HYNIC-PEG** |  |  |  |  |
| N1 | 11.40 | 10.33 | 0.91 | 0.59 |
| N2 | 10.00 | 8.96 | 0.90 | 0.58 |
| N3 | 10.00 | 8.06 | 0.81 | 0.53 |
| L1 | 7.46 | 8.13 | 1.09 | 0.71 |
| L3 | 10.00 | 5.11 | 0.51 | 0.33 |
| L3 | 4.86 | 2.84 | 0.58 | 0.38 |
| **Mean** | **8.95** | **7.24** | **0.80** | **0.52** |
| **SD** | **2.38** | **2.75** | **0.22** | **0.14** |
| **^99m^Tc-LLP2A-HYNIC** |  |  |  |  |
| N1 | 0.99 | 6.84 | 6.91 | 0.57 |
| N2 | 0.56 | 3.82 | 6.82 | 0.56 |
| N3 | 1.28 | 8.52 | 6.66 | 0.55 |
| L2 | 0.93 | 7.58 | 8.13 | 0.67 |
| L2 | 1.27 | 9.00 | 7.09 | 0.59 |
| L4 | 1.00 | 10.68 | 10.68 | 0.88 |
| L5 | 1.90 | 8.55 | 4.50 | 0.37 |
| L5 | 2.40 | 9.24 | 3.85 | 0.32 |
| **Mean** | **1.29** | **8.03** | **6.83** | **0.56** |
| **SD** | **0.59** | **2.05** | **2.10** | **0.17** |

Table S4. Specific activity, injected activity, and injected mass of injected compound of normal dogs N1-N3, and NHL dogs L1-L5.
